# Investigation of the relationship between the onset of arthritis and uveitis in genetically predisposed SKG mice

**DOI:** 10.1186/s13075-015-0725-z

**Published:** 2015-08-19

**Authors:** Ellen J. Lee, Emily E. Vance, Brieanna R. Brown, Paige S. Snow, Jenna S. Clowers, Shimon Sakaguchi, Holly L. Rosenzweig

**Affiliations:** Department of Ophthalmology, Oregon Health & Science University, Portland, OR USA; VA Portland Health Care System, Portland, OR USA; School of Medicine, Oregon Health & Science University, Portland, OR USA; Osaka University, Suita, Osaka Japan; Department of Molecular Microbiology & Immunology, Oregon Health & Science University, Portland, OR USA

## Abstract

**Introduction:**

Systemic rheumatic conditions are often accompanied by intraocular inflammatory disease (termed *uveitis*). Despite the frequent manifestation of uveitis with arthritis, very little is understood of the underlying mechanisms that mediate the eye’s susceptibility to disease. The genetically susceptible SKG mouse strain develops arthritis that arises from an inherent mutation that disrupts T-cell antigen receptor signal transduction and thymic selection. The ensuing T-cell–mediated disease is further modulated through exposure to microbial triggers. The purpose of this study was to elucidate how a genetically determined shift in the T-cell repertoire toward self-reactive T cells that drive arthritis influences uveitis in SKG mice.

**Methods:**

SKG mice (BALB/c mice that harbor the W163C point mutation in zeta-chain-associated protein kinase 70 [i.e., ZAP-70]) were housed under arthritis-resistant, specific pathogen–free conditions. Arthritis was induced by intraperitoneal injection with fungal glucans (zymosan or curdlan). Arthritis onset and severity were evaluated by clinical scoring, histopathology and infrared imaging within the joints. Periocular traits involving blepharoconjunctivitis were evaluated by clinical scoring and histology. Eyes were evaluated for signs of anterior uveitis using intravital videomicroscopy to document cell-trafficking responses within the iris vasculature and stroma and by histology to detect inflammatory infiltrate and tissue damage within the anterior and posterior eye segments.

**Results:**

Exposure to zymosan resulted in the predicted arthritic, sexually dimorphic phenotype in SKG mice. The eyes of SKG mice exhibited episodic intravascular cellular responses to zymosan or curdlan as indicated by significant increases in leukocyte–endothelium interactions akin to ocular vasculitis. However, despite the significant increase in early cell-trafficking responses, cellular infiltration into the iris stroma was not observed and histopathological signs indicative of a sustained uveitis were absent. Instead, eyes of SKG mice developed blepharoconjunctivitis that coincided with arthritis and exhibited sexual dimorphism.

**Conclusions:**

This study underscores the complexity surrounding the pathogenesis of uveitis and its relationship with arthritis. The findings suggest that distinct mechanisms exist by which pathogenic autoimmune T cells target the eyes versus joints, which likely involves the environmental context but nonetheless should be taken into account in the identification and development of effective therapies for each organ.

**Electronic supplementary material:**

The online version of this article (doi:10.1186/s13075-015-0725-z) contains supplementary material, which is available to authorized users.

## Introduction

A number of systemic rheumatic conditions, such as ankylosing spondylitis, psoriatic arthropathy, Behçet disease, juvenile idiopathic arthritis, sarcoidosis, reactive arthritis/Reiter syndrome and Blau syndrome, are commonly associated with intraocular inflammatory disease, or *uveitis*. Uveitis itself encompasses a heterogeneous group of immune-mediated disorders that may also occur in isolation and whose etiology may be infectious or non-infectious (i.e., autoimmune) [1, 2]. The underlying mechanisms of uveitis are poorly understood; however, it is interesting to speculate whether ongoing systemic rheumatic disease and increased frequency of autoreactive T cells that target specific systemic organs and tissues share commonalities with T cells that traffic to the eyes, which would render them susceptible to subsequent ocular disease.

One prevailing theory is that chronic inflammatory diseases such as arthritis and uveitis arise from complex interactions between genetic predisposition and environmental triggers. The role of adaptive immunity, particularly of T cells, in uveitis has traditionally been the subject of intense investigation [3–7]. This is in large part due to the strong genetic association with human leukocyte antigen (HLA) [8–10], which is thought to be involved in aberrant antigen presentation by the major histocompatibility complex and subsequent development of autoreactive T-cell responses. The presence of autoreactive T cells has been a consistently reported feature of uveitis in patients, and pharmacologic modulation of T-cell effector functions can have beneficial effects [11, 12]. Given that T cells are considered important cellular mediators of many arthritic diseases, one may reason that their aberrant autoreactive responses also contribute to coincident uveitis.

To address whether thymic production of pathogenic autoimmune T cells could be a primary cause of ocular disease, we undertook studies in the genetically predisposed arthritic SKG mice. SKG mice, first described by Sakaguchi and colleagues [13], develop self-reactive T cells as a consequence of a point mutation in the Src homology 2 domain of zeta-chain-associated protein kinase 70 (ZAP-70), a critical mediator of T-cell receptor signaling responses. This mutation impairs T-cell signaling capacity and consequently disrupts positive and negative selection in the thymus [14], resulting in the survival of self-reactive T cells that would otherwise have been deleted. A chronic and progressive polyarthritis develops, which also coincides with features of systemic inflammation such as increased autoantibody titers and increased levels of cytokines, including interleukin (IL)-1, IL-6 and tumor necrosis factor α [13, 15, 16]. The arthritis aspect of disease is transferable by CD4^+^ T cells, and the type 17 helper T-cell (Th17) effector response is considered a driving pathogenic response [17]. Extraarticular manifestations, including interstitial pneumonitis, vasculitides, subcutaneous necrobiotic nodules and colitis [13, 15, 16, 18], have also been described. A recent report by Ruutu et al. [19] was particularly interesting and exciting to us in that SKG mice that had received systemic injections with fungal cell wall components such as β-glucan developed anterior uveitis. Thus, generation of an autoreactive T-cell population could affect other target organs, such as the eye.

An interesting caveat to disease development in SKG mice is the prerequisite of an environmental trigger. It has been observed that SKG mice do not develop arthritis in microbially clean housing environments [13, 20]. This indicates that the genetic predisposition causing persistent autoreactive T cells is not sufficient, but that a second signal is required for disease. Indeed, the disease phenotype can be reproduced when SKG mice are exposed to microbial stimuli such as the fungal cell wall components zymosan or β-glucan curdlan [20, 21]. Thus, even though this is a T-cell–mediated disease, environmental factors are a qualification. This is an important distinction from other experimental models of autoimmunity, wherein a break in tolerance is caused by immunization with self-proteins. For example, in experimental autoimmune uveitis, rodents immunized with retinal self-antigens develop autoimmunity targeted to the posterior eye [22–24]. Thus, disease modeled in SKG mice aptly suits the purpose of this study because it is a T-cell–intrinsic disease arising from a break in central tolerance involving environmental cues.

In this study, we tested the hypothesis that generation of an autoreactive T-cell population that drives arthritis in SKG mice is an underlying factor in the development of uveitis. We observed the predicted onset and progression of arthritis in SKG mice exposed to zymosan or curdlan, which occurred more rapidly in females than in males. The generation of the autoreactive T-cell repertoire that gives rise to arthritis, however, did not translate to sustained uveitis, despite the ongoing dynamic cellular responses within the iris vasculature. Interestingly, arthritis instead coincided with blepharoconjunctivitis that followed a similar sexual dimorphism. The observations in this study highlight the complexity surrounding immunological mechanisms by which T cells are involved in uveitis and the manner in which autoreactive T cells target the eye.

## Methods

### Mice

SKG mice on the BALB/c background were maintained under specific pathogen–free (SPF) conditions at the VA Portland Health Care System, Portland, OR, USA. All studies were carried out in accordance with the National Institutes of Health Guide for the Care and Use of Laboratory Animals and with approval granted by the VA Portland Health Care System Institutional Animal Care and Use Committee. Genotype was confirmed as previously described [13]. Briefly, DNA was isolated from tail tissue, and the region of the *Zap-70* gene containing nucleotide 489 (site of G-to-T substitution in SKG mice) was amplified by PCR. Restriction enzyme digestion with BstNI was performed on the PCR product, which cleaved only wild-type (WT) DNA. The resulting products were visualized by agarose electrophoresis. All experiments were carried out in accordance with institutional guidelines for animal welfare.

### Induction and clinical grading of arthritis

SKG mice (7–8 weeks of age) were administered either a single intraperitoneal (i.p.) injection of 3 mg of zymosan, a cell wall component prepared from *Saccharomyces cerevisiae*, or a β-1,3-glucan, curdlan, prepared from *Alcaligenes faecalis* (InvivoGen, San Diego, CA, USA). After injection, the mice were evaluated as a function of time for the onset and severity of arthritis using a previously described grading system adapted for this model [20, 25]. Arthritis was scored on a weekly basis by a masked observer using the following defined criteria: 0 = no joint swelling or redness, 1 = any sign of arthritis (i.e., swelling or redness in any single digit of the wrist or ankle), 2 = mild swelling or redness in wrist and/or ankle coinciding with two or more swollen digits, 3 = severe redness and/or swelling in all digits and throughout the wrist or ankle with reduced ability to grip, and 4 = deformities of fingers and severe ankylosis extending from wrist and/or ankle up to larger joints. Mice were scored for all paws, and scores were summed per mouse such that the clinical grade for a single mouse could range from 0 to a maximum of 16. For calculation of disease incidence, a mouse was considered positive for arthritis when a total clinical score was ≥1 maintained for ≥2 weeks.

### Scoring of periocular manifestations

Periocular changes surrounding the eyeball (e.g., eyelid inflammation [blepharitis] or conjunctival discharge [indicating conjunctivitis]) were features observed coincidentally upon disease induction. Owing to the lack of an established system for scoring severity of these individual characteristics, a binary scoring system was applied to indicate the presence of blepharitis (score of 1); the presence of blepharitis coinciding with conjunctivitis, more severe eyelid swelling and/or ocular discharge (score of 2); or the absence of any inflammation (i.e., normal-appearing eye as in naive BALB/c controls) (score of 0). Mice were scored individually for each eye, and scores were summed per mouse such that the clinical grade for a single mouse could range from 0 to a maximum of 4.

### Histological evaluation

At the indicated times, the eyes and ankles of each animal were collected, fixed in 10 % neutral-buffered formalin and prepared for tissue sectioning as previously reported [26, 27]. Histological assessment of the ankle joints was performed on decalcified ankles. The eyes were embedded in paraffin, sectioned (7 μm) through the pupillary–optic nerve axis and stained with hematoxylin and eosin. At least three sections obtained at four different depths within each eye were scored in a masked fashion, and the number of infiltrated leukocytes present in the aqueous humor of the anterior segment and vitreous body of the posterior segment was quantified.

### Infrared in vivo imaging

Upon termination of the experiment, imaging was performed on legs and spines dissected from animals that had been given ProSense (NEV10003; PerkinElmer, Waltham, MA, USA) injections for detection of areas of signal localization and activation within the joints as previously described [26, 28]. Hair and skin were removed from the legs and spines for optimal signal detection, then scanned with 685-nm and 785-nm lasers and white light-emitting diodes using the Pearl Imager (model 5700; LI-COR Biosciences, Lincoln, NE, USA). Images of matched pairs of light sources were acquired by using a charge-coupled device detector and Pearl Cam software (LI-COR Biosciences), which normalized acquisitions so that the same intensity value was displayed with the same color and grayscale value on all images. Regions of interest (ROIs) were analyzed using Image Studio software (LI-COR Biosciences) and the small animal image analysis application. The mean differences in infrared signaling in the ROIs were normalized to those of corresponding ROIs from healthy, naive WT-BALB/c mice.

### Intravital videomicroscopy

The in vivo cellular trafficking response within the iris vasculature and tissue was evaluated every 2 weeks using intravital videomicroscopy as previously described [26, 27]. At the time of imaging, mice were anesthetized with isoflurane and leukocytes were visualized with rhodamine 6G (35 mg/kg; Sigma-Aldrich, St. Louis, MO, USA), a dye taken up by all leukocytes when administered via i.p. injection. Digital videos (10 seconds each) were acquired in three independent regions of the iris using a monochrome camera (Kappa optronics, Gleichen, Germany). Measurements (diameter and length) of vessels and stromal tissue area, as well as quantification of rolling, adherent and infiltrating leukocytes, were performed offline by masked observers using ImageJ software (National Institutes of Health, Bethesda, MD, USA). Both eyes of each mouse were evaluated.

### Statistics

The results are presented as the mean ± SEM. Statistical analysis of differences between experimental groups was performed by using analysis of variance or two-tailed Student’s *t* tests. For nonparametric data (i.e., clinical arthritis scores or binary periocular scores), the Mann–Whitney *U* test was used (Prism; GraphPad Software, La Jolla, CA, USA). Statistical significance was set at *p* < 0.05.

## Results

### Exposure to zymosan results in chronic and progressive polyarthritis in genetically predisposed SKG mice

**Fig. 1:**
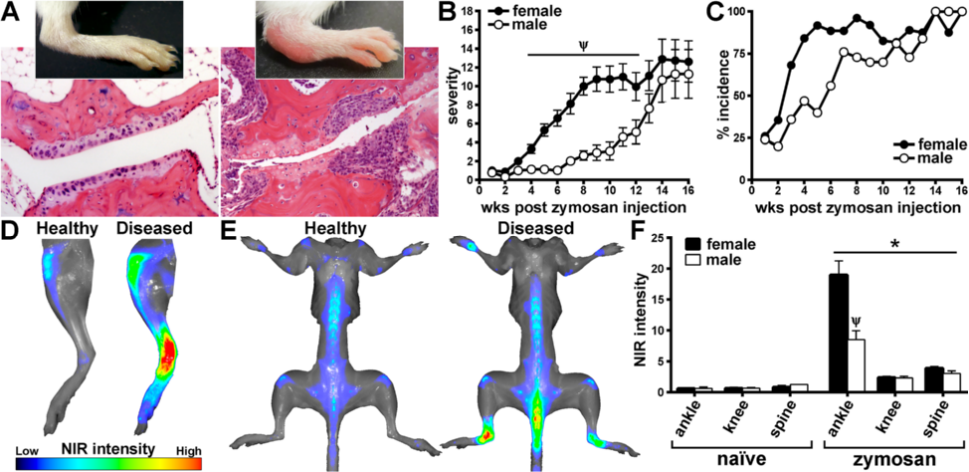
Zymosan exposure results in chronic and progressive polyarthritis in SKG mice. SKG mice were administered a single intraperitoneal injection of zymosan at 7–8 weeks of age. Animals were subsequently evaluated clinically for arthritis every week to 16 weeks after zymosan injection (i.e., 23–24 weeks of age). **a** Representative photographs depict healthy, uninflamed ankle and digit within the ankle of a naive SKG animal (clinical grade 0, *left*) and an inflamed, arthritic ankle (clinical grade 4, *right*) of an SKG mouse that received a zymosan injection. Below the photographs are stained histopathological sections obtained at 16 weeks after zymosan injection. Clinical arthritis evaluation revealed increased severity and faster onset in zymosan-challenged females compared with males (**b**, **c**). Naive SKG mice of either sex did not show any arthritis development (data not graphed). **d** Visualization of deposition and activation of labeled near-infrared (NIR) probe within the legs at 16 weeks after zymosan injection. Knees and ankle and tarsometatarsal joints of legs obtained from a naive BALB/c mouse (Healthy) and an arthritic SKG mouse that had received zymosan injection (Diseased). **e** Whole-body NIR images illustrate changes to the base of the spine occurring at 16 weeks after zymosan injection in a diseased (after zymosan injection) SKG mouse (*right*) versus a healthy, naive BALB/c mouse (*left*). **f** Quantification of regions of interest of SKG mice using NIR imaging technology, wherein all values were normalized to background signaling from naive BALB/c mice. *p < 0.05 indicates effect of zymosan versus naive SKG mice; ^Ψ^ *p* < 0.05 sex comparison between SKG mice that received a zymosan injection; n = 12–18 mice/group (combined two individual experiments). A significant response was observed for both male and female mice that received zymosan injections compared with the non-treated group for onset and severity of arthritis (**b**, **c**)

Consistent with prior reports [20], SKG mice housed under the SPF conditions at our facility remained healthy and did not spontaneously develop overt signs of arthritis (data not shown). However, the reported arthritis phenotype was reproduced with an acute exposure to microbial stimuli such as the β-glucan–containing and fungus-associated product zymosan [20, 21]. As shown in Fig. 1, systemic exposure to zymosan at 7–8 weeks of age resulted in the development of a progressive polyarthritis in SKG mice within the ankles and wrists, which translated histologically into thickening of synovial lining and infiltration of sublining coincident with bone and cartilage destruction and pannus formation (Fig. 1a). The female SKG mice developed earlier onset and more severe arthritis compared with males (Fig. 1b, c), further confirming the sex × gene interaction in the disease phenotype of SKG mice [16, 29]. Ultimately, both sexes achieved a near 100 % incidence of arthritis (i.e., 16 weeks postinjection) (Fig. 1c). Using infrared imaging technology, which we have found closely predicts the extent of joint pathology [27, 30], we were able to more sensitively quantify inflammatory arthritis within the joints, thereby showing significant inflammation within the knee that extended down the tibiotarsus to the ankle and tarsometatarsal joints (Fig. 1d). Some inflammation was detected within the lower spine as well, wherein positive signals localized to the vertebral column just below the sacrum and within the upper caudal vertebrae of the tail (Fig. 1e). Quantification of changes in localized infrared signals compared with naive BALB/c mice (Fig. 1f) showed minimal to undetectable signaling in the naive SKG mice, but signaling was significantly enhanced upon zymosan exposure in both sexes. It was noted, however, that the ankle and/or tarsometatarsal joints demonstrated the most robust inflammatory response to zymosan versus the knee or spine, thereby indicating a predilection for smaller synovial joints, such as condyloid and saddle joints, in their susceptibility to this type of disease.

### Development of blepharoconjunctivitis coincides with arthritis in SKG mice

**Fig. 2:**
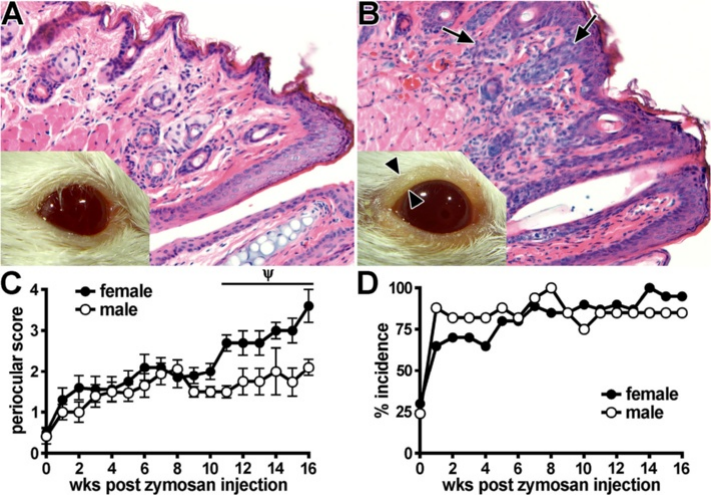
Evaluation of blepharoconjunctivitis development in SKG mice. **a** Photograph and stained histological section of a naive, healthy control BALB/c mouse. **b** Photograph and stained histological section of an SKG mouse that had received a zymosan injection, showing eyelid inflammation (blepharitis) and conjunctival discharge. *Arrowheads* indicate thickened lid margin, which coincides with epithelial hyperplasia and leukocytic infiltration visualized by histopathology (*arrows*). The presence of such periocular manifestations in SKG mice was graded over time (0–16 weeks after zymosan injection) using a binary scoring system to assay for the complete absence (score of 0 as shown in panel **a**), the presence of blepharitis (score of 1) or blepharitis with coinciding conjunctivitis and/or discharge (as shown in panel **b**; score of 2). Total score per mouse (sum of both eyes) (**c**) and percentage incidence (score ≥1; panel **d**) were plotted as a function of time since zymosan injection. A prominent response (*p* < 0.05) was observed for both male and female mice that received a zymosan injection versus SKG mice that did not receive a zyomsan injection (**c**). ^Ψ^ *p* < 0.05 indicates effect of sex among SKG mice that received a zymosan injection; n = 12–18 mice/group (combined two individual experiments). BALB/c mice were included as a negative control group (scores = 0)

We observed an obvious periocular response to zymosan that manifested initially as eyelid inflammation (i.e., swelling and redness similar to blepharitis). With zymosan, this feature was markedly enhanced and progressed to involve massive cellular infiltration in and around the meibomian glands (Fig. 2a, b). Such pathological changes coincided with mucous discharge on the ocular surface, as is typical in conjunctivitis. Evaluating the onset of this blepharoconjunctivitis as it relates to arthritis progression using basic, binomial criteria, we found that the milder form of blepharitis, which localized to the posterior eyelid, occurred spontaneously in approximately 25 % of SKG mice compared with 0 % in healthy BALB/c control mice (data not shown). With zymosan exposure, the presence of blepharoconjunctivitis in at least one eye preceded the clinical onset of arthritis and progressed throughout the course of disease (Fig. 2c, d). Interestingly, blepharoconjunctivitis also followed a similar sexual dimorphism in that females demonstrated a more severe condition (Fig. 2c). These data suggest an autoimmune, T-cell–intrinsic component as an underlying mechanism of blepharitis and/or conjunctivitis that could be akin to prior reports of skin inflammation [13, 19].

### Disease triggered by zymosan in SKG mice results in dynamic cellular responses within the vasculature of the eye

**Fig. 3:**
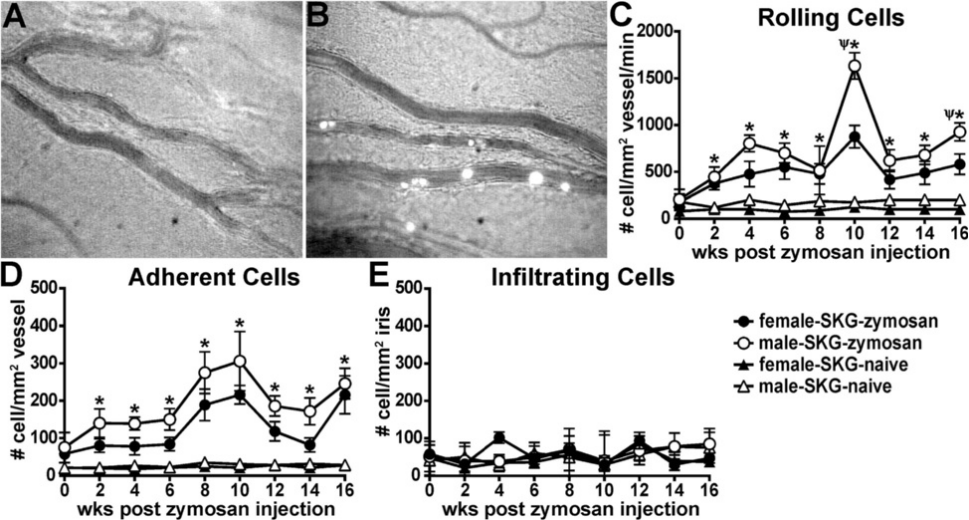
Intravital videomicroscopy reveals cellular rolling and adhesion, but not infiltration, within the microvasculature of the eye. Photographs show representative still images from videomicroscopy of the iris vasculature and rhodamine-labeled hematopoietic cells from uninflamed eyes of naive SKG mice (**a**) and from inflamed eyes of SKG mice that had received a zymosan injection (**b**). The iris vasculature and tissue were imaged biweekly over a 16-week period in naive SKG mice or in SKG mice that had received a zymosan injection, and videos were analyzed offline for numbers of rolling, adherent and infiltrated cells per square millimeter of vessel or iris tissue (**c**–**e**). *p < 0.05 indicates comparison between zymosan and naive SKG mice within each sex; Ψ indicates sex effect within SKG mice that had received a zymosan injection; n = 12–18 mice/group (combined two individual experiments)

Given the overt periocular manifestations (Fig. 2) and a prior report of the presence of an anterior uveitis in SKG mice [19], we sought to further evaluate the onset and kinetics of intraocular inflammation in arthritic SKG mice. Using intravital videomicroscopy, we performed a thorough examination of cell-trafficking responses within the iris, which is the tissue consistently affected by anterior uveitis. Intravital videomicroscopy is a reliable and sensitive technique that enables not only quantification of cells within the vessels and tissues of the iris but also ascertainment of dynamic behavioral changes longitudinally over time in their rolling, adhesion or infiltrative properties that can result in ocular damage [26, 27, 30]. Such behaviors are exemplified in the representative still images shown in Fig. 3a, b, which are derived from videomicroscopy movies that show this in more detail (Additional files 1 and 2). Naive SKG mice did not spontaneously develop any intraocular inflammation, as shown by negligible cellular responses involving leukocyte–endothelium interactions (Fig. 3a, c–e,) akin to those in healthy, uninflamed eyes of BALB/c mice. However, with zymosan injection, a marked increase in leukocyte rolling and adhesion along the endothelium was observed (Fig. 3b–d). Quantification from video data showed the significant rolling and adhesion cellular responses that were increased with zymosan exposure (Fig. 3c, d), which peaked around 10 weeks and again at 16 weeks (Fig. 3c, d). This cellular response involving leukocyte–endothelium interactions is likely indicative of an ocular vasculitis that ensues upon endothelial cell activation and upregulation of adhesion molecules (e.g., selectins, integrins) that are known to mediate such cellular interactions. Notably, this response was detectable before clinical arthritis onset, and, unlike the progressive nature of arthritis, these ocular cellular changes were episodic in that they followed a phasic response.

**Fig. 4:**
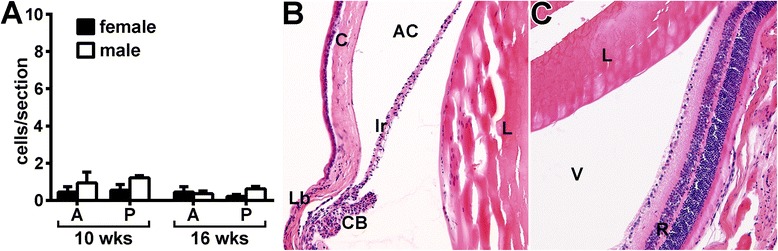
Histopathological evaluation reveals absence of uveitis in animals that had received a zymosan injection. Eyes of SKG mice were evaluated at 10 weeks or 16 weeks after zymosan injection for signs of ocular disease. The number of leukocytes within the aqueous humor of the anterior segment and within the vitreous body of the posterior segment in response to zymosan was quantified (**a**). Representative photographs show the anterior (**b**) and posterior (**c**) eye segments at 10 weeks after zymosan injection. *A* anterior, *P* posterior, *AC* anterior chamber, *C* cornea, *Ir* iris, *Lb* limbus, *CB* ciliary body, *L* lens, *V* vitreous, *R* retina. Statistical significance was not achieved for zymosan versus naive SKG mice of either sex at either time point. n = 17–23 mice/group or versus BALB/c controls (combined three individual experiments)

Interestingly, such early intravascular cell-trafficking responses did not appear to lead to increased cellular infiltration into the iris stroma (Fig. 3e). We further evaluated ocular inflammation by histology at both 10 weeks and 16 weeks after zymosan injection, which had appeared by intravital videomicroscopy to be “peak” time points for intravascular responses. As shown in Fig. 4, histopathological examination did not reveal any significant features of sustained ocular disease. The number of leukocytes quantified within the aqueous humor of the anterior segment and the vitreous body of the posterior segment was negligible (Fig. 4a) and statistically indistinguishable from the number in naive SKG controls or naive BALB/c mice (data not shown). Figure 4 shows representative images of anterior (Fig. 4b) and posterior (Fig. 4c) eye segments from an arthritic animal at 10 weeks following zymosan exposure. Consistent with the intravital data (Fig. 3) indicative of vasculitis, some engorgement of iris vessels was noted. However, other signs present during clinical uveitis, such as fibrin deposition within the chambers, keratic precipitates and optic nerve inflammation, were not observed in diseased SKG mice. These data indicate that, despite the ongoing leukocyte–endothelium interactions occurring within the iris microvasculature of SKG mice, uveitis as defined pathologically did not develop.

### Curdlan-triggered disease in SKG mice results in arthritis and intra-vasculature inflammatory responses within the eye

**Fig. 5:**
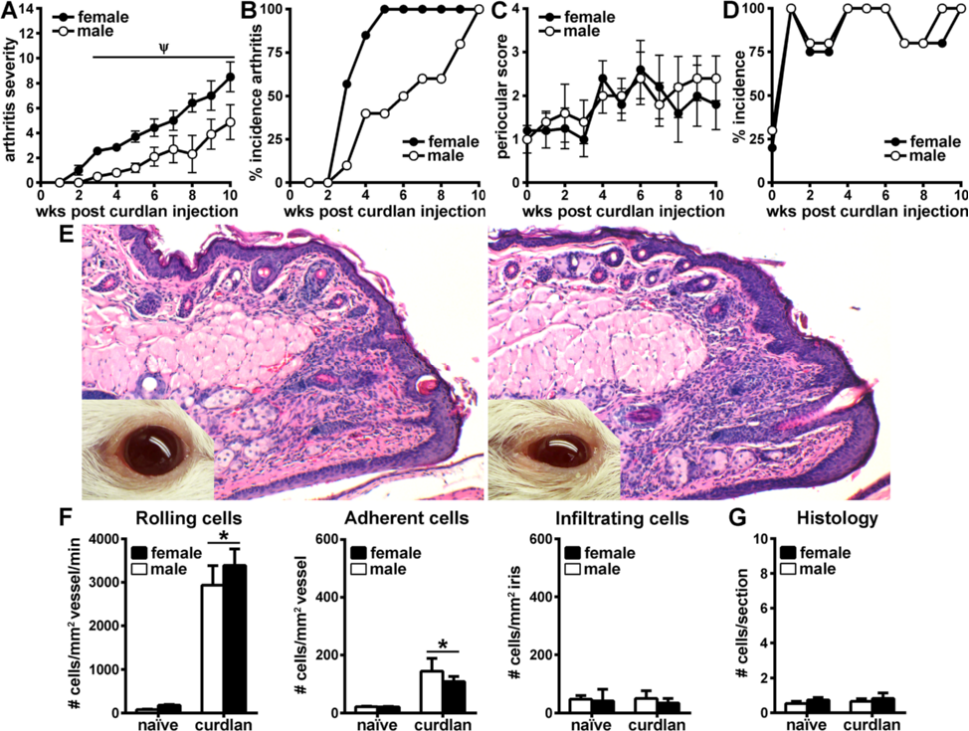
Curdlan exposure reproduces arthritic phenotype in SKG mice but does not result in uveitis. Animals evaluated clinically for arthritis for 10 weeks following curdlan injection exhibited increasingly severe arthritic disease (**a**) and increased incidence (**b**) over time, with females showing worse disease and earlier onset than males. **c–e** The onset and severity of periocular manifestations in SKG mice were graded in response to curdlan using the binary scoring system (as in Fig. 2) and images of the presence of blepharitis (score of 1; **e**, *left*) or blepharitis with coinciding conjunctivitis and/or discharge (score of 2; **e**, *right*). **f** Analysis of intravital videomicroscopy of iris vasculature and tissue at 10 weeks following curdlan injection revealed significant increases in intravascular leukocyte rolling and adhesion in curdlan-injected animals compared with naive SKG mice. These increases were not accompanied by leukocyte infiltration into extravascular iris tissue. **g** Quantification of eye histological sections from naive versus curdlan-injected animals at 10 weeks show no difference in the number of cells infiltrating into the anterior and posterior chambers in curdlan-injected mice. *p < 0.05 for comparison between curdlan-injected and non-injected SKG mice (observed in both sexes); ^Ψ^ *p* < 0.05 indicates comparison between sexes in curdlan-injected SKG mice; n = 12 mice/group (combined two individual experiments)

In addition to zymosan, purified β-glucans such as curdlan have been described as potent triggers of arthritis in SKG mice and as having periocular disease manifestations [20]. Thus, to further evaluate the discordant disease responses we observed in the joints and eyes of SKG mice injected with zymosan, we reevaluated uveitis and arthritis elicited in response to curdlan. Similarly to zymosan-injected animals, systemic curdlan injection reproduced the arthritic, sexually dimorphic phenotype in SKG mice (Fig. 5a, b). We found that a prior exposure to curdlan resulted in development of progressive polyarthritis in SKG mice (Fig. 5a, b), albeit to a slightly lesser degree compared with zymosan (Fig. 1). The female mice developed an earlier onset and a more severe form of arthritis than did males, but 100 % incidence occurred by 10 weeks in both sexes (Fig. 5b). We found that prior exposure to curdlan elicited a periocular response akin to that with zymosan, albeit to a lesser degree of severity in its clinical manifestations (Fig. 5c–e). Analysis of intravital microscopy videos of curdlan-injected SKG mice revealed dramatic intravascular leukocyte dynamics indicative of a vasculitis response at 10 weeks that involved leukocyte rolling and adhesion along the endothelium (Fig. 5f). However, such cellular interactions did not lead to iris stromal tissue infiltration (Fig. 5f). This observation was confirmed by histological evaluation, which showed an absence of cells within ocular tissue or the chambers (Fig. 5g). These findings indicate that zymosan and the purified β-glucan curdlan are both potent triggers of coincident periocular inflammation and arthritis but do not induce uveitis.

## Discussion

On the basis of a prior report [19], we sought to further elucidate how generation of a self-reactive T-cell population that causes arthritis confers susceptibility to uveitis. We confirmed the sex × gene interaction in arthritis development in the SKG mouse strain in response to both zymosan and curdlan. Moreover, we expanded upon the description of the coincident periocular disease that occurred in arthritic SKG mice, which indicates the involvement of self-reactive T cells in the eyelid and/or conjunctival inflammation. This may be a relevant observation because psoriasis-like skin inflammation is a sign common to many rheumatic diseases in which arthritis also develops. Blepharitis (i.e., eyelid inflammation) can lead to dry eye syndrome, which in some forms is believed to have an autoimmune component. Despite such periocular manifestations in our mice, however, our data do not support the hypothesis that a common autoreactive T-cell population causes both arthritis and uveitis. We observed dynamic cellular responses within the vasculature of the eye that were indicative of episodic vasculitis, but these ongoing cellular responses did not lead to infiltration into the iris tissue or any other histopathological sign of uveitis.

Our findings reveal a significant increase in leukocyte–endothelium interactions within the iris vasculature that is likely indicative of vasculitis. This response peaked around 10 weeks after zymosan injection, then decreased and peaked again at 16 weeks. This episodic pattern is reminiscent of episodic flares that are known to occur clinically in patients with anterior uveitis. Such flares could be indicative of changes in vascular permeability that we were unable to detect at the cellular level and/or of endothelial activation resulting from spikes in serum levels of cytokines, although the progression and/or kinetics of cytokine responses in the circulation needed to determine this have not been fully characterized. Such acute immune responses could also be caused by environmental and/or microbial triggers elicited by microbiota that are known to influence systemic immune responses. Ultimately, endothelial dysfunction caused directly or indirectly as a bystander effect of systemic autoimmunity is very relevant clinically, as increased morbidity is associated with systemic diseases such as rheumatoid arthritis. In other words, generalized inflammation that damages blood vessels can potentiate or aggravate diseases such as atherosclerosis and stroke.

Using two different methodological approaches (intravital videomicroscopy and histopathology), we were unable to detect any signs of intraocular inflammation that could be classified as clinical uveitis. This suggests that the blood–ocular barrier remained intact even in the face of ongoing systemic inflammation and vascular dysfunction. This finding indicates that autoreactive mechanisms driving T-cell–mediated disease targeted to the joint, skin or vasculature may differ from those of the eye and/or that additional signals are required for complete expression in the eye for uveitis to develop. Thus, we cannot conclude that a T-cell–intrinsic defect that causes arthritis also leads to uveitis. This may be analogous to the case of HLA-B27. HLA-B27 is strongly associated genetically with spondylarthritis, particularly with ankylosing spondylitis and associated acute anterior uveitis (AAU). For example, possession of HLA-B27 antigen increases the relative risk of AAU by 26 times [10, 31, 32]. Animal models of HLA-B27–associated disease have clearly demonstrated the direct connection between HLA-B27 expression and multisystem inflammatory disease involving spondylarthritis [33, 34]; yet, uveitis is not an observed spontaneous phenotype in the HLA-B27 transgenic animal models [35, 36]. Therefore, what is sufficient for arthritis induction is not necessarily sufficient for uveitis development. The dissonant responses among the eyes and joints are exemplified also in a murine model of spondylarthritis induced by active immunization with proteoglycan [25, 27]. Arthritis is mediated by a Th1-dependent mechanism; yet, deficiency in interferon γ worsened the uveitis aspect of disease [27], thereby indicating that a discordant mechanism involving Th1 effector responses can exist between the eyes and joints (i.e., despite being target organs in the same disease). Thus, the same mechanisms do not necessarily have similar effects in the eyes and joints.

In a recent study of SKG mice, Ruutu and colleagues reported anterior uveitis in approximately 25 % of diseased SKG mice upon β-glucan exposure [19]. In our present study, in which we examined ocular disease within the same time frame as their study, we were unable to recapitulate this particular phenotype of disease induced in SKG mice. However, we were able to consistently reproduce other reported features of disease induced in SKG mice by fungal components, such as the periocular manifestations and lung-associated pneumonitis (data not shown), which is known to occur in approximately 100 % of the animals and is believed to be caused by the same T-cell clone as in arthritis [18]. Intriguingly, in the prior report [19], Ruutu and colleagues did not observe the lung-associated disease, suggesting that different disease penetrance within the SKG mice may occur between the two animal facilities. Another phenotypic difference noted between the two studies is the extent of axial disease. Consistent with the prior report [19], our studies reveal ongoing inflammation within the lower vertebral column, just below the sacrum and within the upper caudal vertebrae of the tail; however, such localized spinal inflammation did not lead to the same extensive inflammatory arthritis compared with that in the smaller synovial joints, such as the ankle and/or tarsometatarsal joints. Such differences in phenotype are intriguing and could relate to different microbial triggers that influence certain aspects of disease. It is conceivably noteworthy that the episodic peaks in ocular vasculitis detected in our experiments coincided with the time frames Ruutu et al. reported for the onset of uveitis. This suggests additional cellular mechanisms not intrinsic to T cells that could participate in underlying mechanisms of uveitis. Another potential consideration is the environmental triggers and/or colony differences related to microbiota colonization in each holding facility. Indeed, in a recent report, researchers observed a similar discordance between arthritis and ileitis incidence under germ-free conditions [37], supporting a link between organ-specific tissue pathology and the microbiota. Collectively, these observations underscore the complexity surrounding extraarticular manifestations such as uveitis in the context of arthritic disease.

## Conclusions

The complexity surrounding uveitis, regardless of whether it is associated with a systemic rheumatic disease, has presented considerable challenges to understanding its pathogenesis. These studies using SKG mice provide proof-of-concept that a break in central tolerance in which autoreactive T cells are generated and can cause chronic and progressive arthritis is not necessarily sufficient for the development of uveitis. T cells clearly play a role in autoimmunity, but the manner in which they are regulated and target the eye are complicated and unlikely to mirror the T-cell responses that occur in the joints. These distinct differences have implications for the identification of safe and effective new therapies that are optimal for the control of immune responses in each organ.

## Additional files

Additional file 1:
**Movie file showing a lack of any active cellular response within the microvasculature of the eye from a naive SKG mouse.** (MOV 15651 kb) Additional file 2:
**Movie file showing the ongoing cellular response within the microvasculature of the eye from a zymosan-challenged SKG mouse.** (MOV 12376 kb)

## Abbreviations

AAU: acute anterior uveitis
HLA: human leukocyte antigen
i.p.: intraperitoneal
IL: interleukin
ROI: region of interest
SH2: Src homology 2
SKG: mouse line derived by S. Sakaguchi that contains genetic abnormality in *Zap-70*
SPF: specific pathogen-free
Th: helper T cell
WT: wild type
ZAP-70: zeta-chain-associated protein kinase 70
